# Inflammable Eructations

**Published:** 1902-10-25

**Authors:** 


### INFLAMMABLE ERUCTATIONS.

Dr. Antony A. Martin,[1] of Eastbourne, reports the case of an elderly man who had been suffering for some weeks from a severe atonic dyspepsia, a marked feature of the case being the fermentative decomposition of the contents of the dilated stomach, with frequent eructations of foul gas, tasting like rotten eggs. One day, two hours after a light breakfast, he was obliged to eructate just as he was lighting his pipe with a match; instantly there was a blinding flash and a slight report due to the ignition of the gases released from the stomach. The patient's beard and eyebrows were thoroughly singed, but no further damage was done. We may presume that the inflammable gas formed in this case was light carburetted hydrogen or marsh gas ($CH_4$), or perhaps simple hydrogen, both of which gases are often found in the intestines and might possibly occur in the stomach when decomposition of the stomach contents has proceeded to an extreme extent. It appears that the intestinal gases vary a good deal according to the food taken. On milk diet hydrogen is the one chiefly formed, whereas on flesh food, and especially on a diet of peas, marsh gas predominates, and may form the major part of the gases discharged from the anus. As is well known marsh gas is a common product of the decomposition of vegetable matter; hence its name.

1 Lancet, Oct. 11.

